# Soil fertility context shapes biodiversity–carbon associations across forest strata in a subtropical karst landscape

**DOI:** 10.3389/fpls.2026.1942459

**Published:** 2026-09-23

**Authors:** Jie Zheng, Jufu Zhang, Meigui Yang, Ailian Zou, Hao Xiong, Muhammad Arif

**Affiliations:** 1 Yunnan Collaborative Innovation Center for Plateau Lake Ecology and Environmental Health, College of Agronomy and Life Sciences, Kunming University, Kunming, Yunnan, China; 2 Key Laboratory of Eco-Environments in the Three Gorges Reservoir Region (Ministry of Education), College of Life Sciences, Southwest University, Chongqing, China; 3 School of Economics and Business Administration, Heilongjiang University, Harbin, China

**Keywords:** biodiversity–carbon relationships, cross-stratum contrasts, forest stratification, multidimensional biodiversity, soil fertility, subtropical forests

## Abstract

Forests in karst landscapes occur across strong edaphic gradients that may alter biodiversity–carbon relationships, yet these relationships are commonly evaluated using tree aboveground biomass carbon, potentially obscuring contrasting responses among forest strata and biomass compartments. We examined how soil fertility context modifies multidimensional biodiversity–carbon associations across aboveground and allometrically estimated belowground compartments in a subtropical karst forest landscape of China. Fifty-four plots were classified as low-fertility (LF) or high-fertility (HF) based on soil nutrient conditions. We quantified tree and shrub aboveground carbon and allometrically estimated root carbon, and assessed plot-level taxonomic diversity, functional diversity, community-weighted trait composition, structural diversity, and abiotic conditions. Associations were evaluated using correlation, regression, relative-importance, and piecewise structural equation analyses. Tree aboveground and estimated tree root carbon were higher under LF conditions, whereas both shrub carbon components were higher under HF conditions. Associations between plot-level biodiversity attributes and carbon stocks varied strongly among woody strata and fertility contexts. Under HF conditions, plot-level species diversity was positively associated with tree carbon but negatively associated with shrub carbon, while structural diversity was positively related to estimated tree root carbon but negatively related to shrub carbon. Under LF conditions, plot-level functional diversity showed negative fitted paths to tree carbon but positive paths to shrub carbon. Predictor rankings and pathway configurations differed across all four carbon compartments, and the measured predictors explained more variation under HF than LF conditions. These findings show that soil fertility is associated with contrasting carbon-storage patterns between tree and shrub strata and that aggregated metrics can conceal reversals in biodiversity–carbon relationships. Resolving forest strata and aboveground and allometrically estimated root-carbon components is essential for understanding plant diversity and carbon storage and informing vegetation restoration in karst landscapes.

## 1 Introduction

Forests occupy approximately 30% of the Earth’s land surface and store about 861 Pg C, nearly 42% of living biomass (Lin et al., 2026; Lunow et al., 2026). Explaining variation in these stocks is central to biodiversity conservation, forest management, and nature-based climate mitigation (Balducci et al., 2026; Maure et al., 2023). Forest carbon is associated with climatic, topographic, and edaphic conditions and with the taxonomic, functional, and structural attributes of plant communities (Lanuza et al., 2026; Zhou et al., 2026). Yet most evidence derives from trees’ aboveground biomass or carbon aggregates across communities (Liu et al., 2025; Zhang et al., 2023b, 2017). This assumes that biodiversity carbon associations are comparable among canopy trees, understory shrubs, aboveground tissues, and roots (Ali et al., 2019; Luo et al., 2019a; Wen et al., 2022). Because these compartments differ in size, structure, canopy position, light access, and rooting depth, an association observed for total or tree aboveground carbon may not characterize shrub or estimated root carbon (Bolstad et al., 2018; Chen et al., 2025; Weigelt et al., 2021). Aggregation may therefore conceal cross-stratum contrasts and shifts in predictor importance (Haq et al., 2024; Luo et al., 2019b; Schnabel et al., 2025).

Resolving woody carbon by stratum and pool addresses this limitation. Trees contain most woody biomass and are associated with canopy dominance, deeper rooting profiles, and pronounced size variation (Li et al., 2026b; Siregar et al., 2026). Shrubs occupy lower canopy positions, depend more strongly on local light and near-surface resources, and contribute to regeneration, nutrient cycling, and understory carbon accumulation (Deng et al., 2023; Geng et al., 2024). Aboveground carbon represents stems, branches, and foliage, whereas root carbon represents belowground biomass estimated allometrically; these components may correspond differently to biodiversity and environmental conditions (Jing et al., 2021; Zhao et al., 2024). Nevertheless, shrub and root carbon remain less frequently examined than tree aboveground biomass or total community carbon. Separating tree aboveground, tree root, shrub aboveground, and shrub root carbon within the same plots, therefore, provides a common basis for identifying cross-stratum and cross-pool contrasts (Schuldt et al., 2023).

A compartment-resolved analysis also requires multidimensional biodiversity metrics (Naka and Fatunsin, 2025; Springer et al., 2024; Wu et al., 2025). Species diversity is commonly interpreted through niche complementarity and selection frameworks (Sonkoly et al., 2019). Complementarity links functional dissimilarity with differentiated resource use, whereas selection links species-rich communities with dominant, high-biomass species (Gao et al., 2021; Noulèkoun et al., 2024). Positive diversity–carbon relationships are expected where these processes predominate, but weak or negative relationships also occur where environmental filtering, functional redundancy, or low-biomass species correspond to limited carbon gains (Bordin et al., 2023; Zheng et al., 2025; Zou et al., 2024). The mass-ratio hypothesis instead emphasizes dominant-species traits, making community-weighted mean traits relevant to resource-use strategies and carbon accumulation (Fatunsin, 2025; Khan et al., 2025; Lin et al., 2016). Structural diversity adds variation in diameter, height, and crown dimensions (Fatunsin and Naka, 2025; Guo et al., 2025; Li et al., 2026a). It may correspond to three-dimensional space occupation and stratified light interception, yet the same configuration may indicate canopy restriction for shrubs (Bourdier et al., 2016; Hwang and Zhai, 2026; Zhai et al., 2024). Height and crown-width variations remain less studied than diameter distributions. Structural diversity may also cross into coexistence and functional diversity (Sun et al., 2025). Integrating taxonomic, functional, trait-compositional, and structural attributes thus permits comparison across carbon components.

Soil fertility provides the environmental context for this comparison (Augusto and Boča, 2022; Gao et al., 2025; Soong et al., 2020). Soil carbon and nutrient availability correspond to plant growth conditions, community assembly, functional strategies, and competitive environments (Mao et al., 2019; van der Sande et al., 2018; Zhang et al., 2023a). At low fertility, environmental filtering often coincides with conservative strategies and greater relevance to dominant trait composition or particular structural attributes (Lin et al., 2023; Ma et al., 2023; Mao et al., 2023). At high fertility, lower nutrient limitations may coincide with greater trait variation, higher understory biomass, and stronger competition for light and space (Chen et al., 2024; Ullah et al., 2026). These settings represent contrasting ecological contexts rather than experimental treatments or formally tested moderators. Soil fertility may correspond with carbon-stock differences and distinct configurations of biotic and abiotic correlates across compartments. Previous research has often considered fertility, diversity dimensions, forest strata, or aboveground–belowground carbon separately (Kazempour Larsary et al., 2025; Wan et al., 2026; Zhao et al., 2026); less attention has been given to their coordinated comparison across the four woody carbon components in subtropical forests. Diversity dimensions may therefore carry different meanings across compartments.

We addressed this gap using subtropical forest plots in China and a parallel, compartment-resolved framework. We analyzed tree aboveground carbon, allometrically estimated tree root carbon, shrub aboveground carbon, and allometrically estimated shrub root carbon separately. Six soil variables classified plots into low- and high-fertility contexts (**Supplementary Figure S1**). High-fertility plots had higher soil carbon and nutrient values than low-fertility plots. Within each context, the same predictor framework, including species diversity, functional diversity, community-weighted trait composition, structural diversity, and abiotic variables, was applied to all four components. We investigated the following: (1) How did the four components vary between fertility contexts? (2) Which diversity and structural attributes were associated with each component, and what cross-stratum contrasts emerged? (3) How did predictor importance and fitted pathway configurations vary among components and contexts? Accordingly, we hypothesized that (H1) woody-carbon responses to soil fertility would differ between tree and shrub strata rather than vary uniformly across the whole woody community; (H2) the same plot-level taxonomic, functional, and structural diversity attributes would show contrasting associations with tree and shrub carbon components because tree and shrub strata differ in resource access and competitive environments; and (H3) the relative importance and pathway configurations of biotic and abiotic predictors would differ between low- and high-fertility contexts. By resolving woody carbon into four components, we evaluated whether biodiversity–carbon conclusions depended on forest stratum, carbon pool, and fertility context.

## 2 Materials and methods

### 2.1 Study area

We established 54 forest plots in Dianjiang County, Chongqing Municipality, southwestern China (29°38′–30°31′ N, 107°13′–107°40′ E; **Figure 1**). The study area occupies a subtropical montane landscape spanning approximately 350–1,129 m above sea level. The climate is humid subtropical monsoon, with a mean annual temperature of 17.1 °C and mean annual precipitation of approximately 1,200 mm. Dianjiang has a geologically heterogeneous setting in which carbonate-bearing formations occur together with non-carbonate lithologies. Karst landforms are locally developed where limestone is exposed, whereas sandstone and other clastic rocks are also widespread. We therefore describe the study setting as a karst-influenced landscape rather than implying that all sampled plots occur exclusively on carbonate-derived substrates. Under the Chinese soil taxonomy, the dominant soils within the sampled forests are montane yellow and yellow-brown soils developed mainly from shale, conglomerate, and sandstone parent materials. The tree layer is dominated by *Pinus massoniana*, *Cunninghamia lanceolata*, and *Cupressus* spp., whereas the shrub layer includes *Rhododendron*, *Pyracantha*, and *Coriaria* spp. Natural variation in soil resources, together with the vertically differentiated woody vegetation, provided a field setting for comparing compartment-specific carbon associations across contrasting fertility contexts (Bolstad et al., 2018; Ullah et al., 2026).

**Figure 1:**
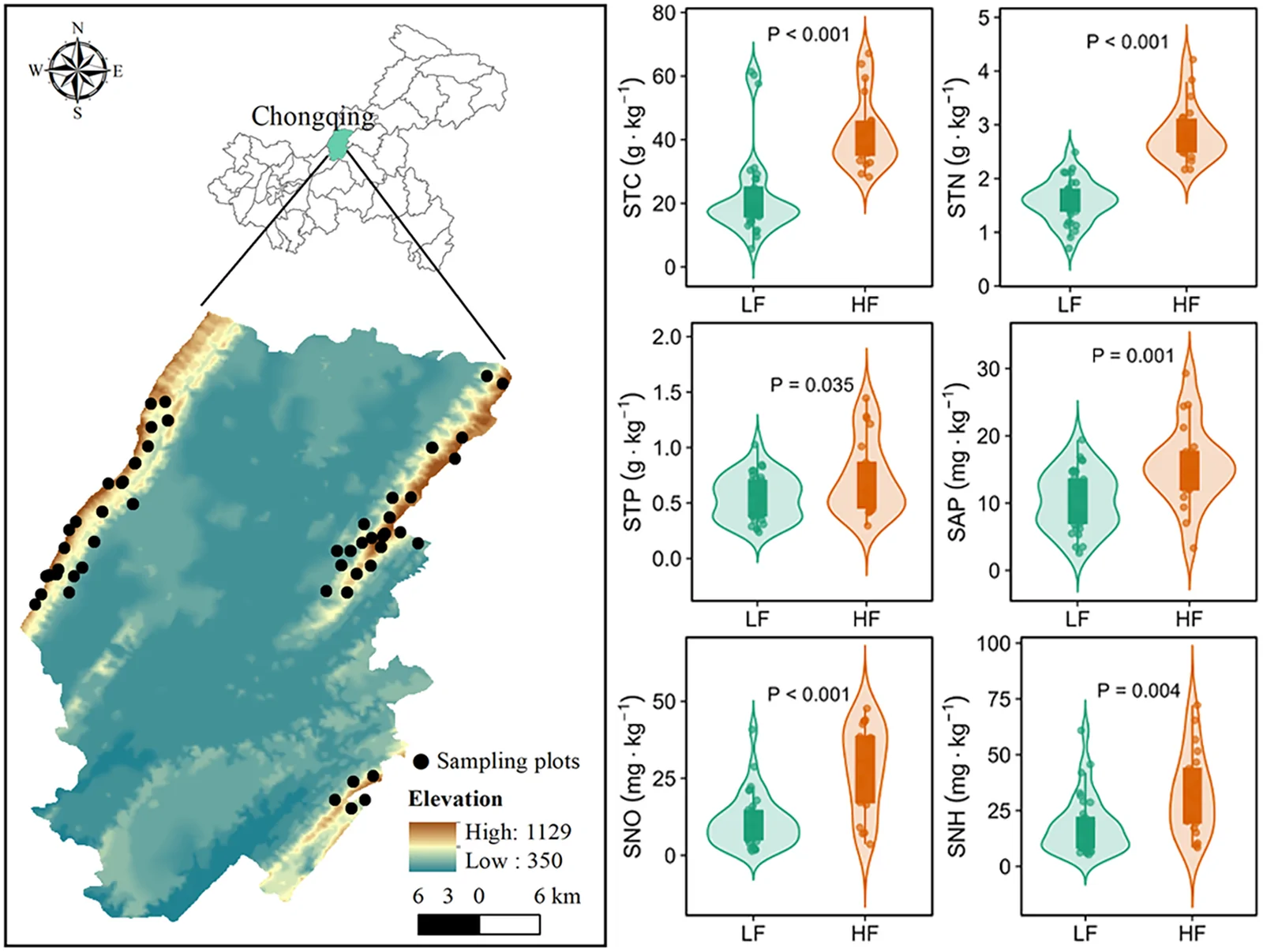
Study area, plot distribution and differentiation of low- and high-fertility forest plots. Violin–box plots compare six soil variables between low-fertility (LF) and high-fertility (HF) plots: soil total carbon (STC), soil total nitrogen (STN), soil total phosphorus (STP), soil available phosphorus (SAP), soil nitrate nitrogen (SNO) and soil ammonium nitrogen (SNH).

### 2.2 Plant and soil sampling

Field surveys were conducted during the peak growing season in 2023. Each 20 m × 20 m plot was divided into four 10 m × 10 m subplots for tree-layer measurements. A 5 m × 5 m shrub quadrat was established within each subplot to survey the understory woody vegetation. This nested design sampled the principal woody strata within the same plot network and allowed tree and shrub carbon stocks to be estimated separately (Ali et al., 2019; Wen et al., 2022; Zhang et al., 2023b). All woody individuals in the tree layer with height ≥3 m and diameter at breast height (DBH) ≥5 cm were identified as species (Gao et al., 2025). DBH, total height, and crown dimensions were measured using standardized field protocols. In the shrub layer, all woody individuals less than 3 m in height were identified, and basal diameter, height, abundance, and cover were recorded (Deng et al., 2023; Geng et al., 2024). Five topsoil sub-samples were collected from the center and four corners of each plot at 0–20 cm depth. The sub-samples were homogenized into one composite sample per plot. Stones, roots, and visible plant residues were removed, after which the samples were air-dried, passed through a 2-mm sieve, and retained for physicochemical analyses.

### 2.3 Soil and environmental variables

Soil pH was measured using a pH meter. Soil bulk density (BD) was determined using the clod method, and soil water content (SWC) was measured by oven drying. Soil total carbon (STC) and soil total nitrogen (STN) were quantified using an elemental analyzer. Soil total phosphorus (STP) was measured by inductively coupled plasma optical emission spectrometry (ICP-OES; ICAP 6000). Soil available phosphorus (SAP) was determined using the Olsen method, soil nitrate nitrogen (SNO) was quantified spectrophotometrically, and soil ammonium nitrogen (SNH) was measured using an intermittent chemical analyzer (Gao et al., 2025; Soong et al., 2020; van der Sande et al., 2018). Altitude was recorded using a handheld global positioning system (GPS) receiver, and slope was measured using a clinometer. Mean annual temperature (MAT) and mean annual precipitation (MAP) were extracted from WorldClim version 2.1 using the geographical coordinates of each plot. STC, STN, STP, SAP, SNO, and SNH were used in cluster analysis to classify the plots empirically into contrasting multivariate soil fertility contexts (Mao et al., 2019, 2023; Zhang et al., 2023a). Prior to clustering, the six soil variables were standardized to z-scores to place them on a comparable scale. Euclidean distances among plots were calculated from the standardized variables, and clustering was performed using Ward’s minimum-variance method (Ward.D2). The resulting two groups comprised 33 low-fertility (LF) plots and 21 high-fertility (HF) plots and were designated according to their contrasting overall soil carbon and nutrient profiles. These categories represented naturally occurring differences in plot-level soil carbon, nutrient pools, and available nutrient supply; they were not experimentally imposed treatments.

### 2.4 Compartment-resolved woody-carbon estimates

Four corresponding woody-carbon components were estimated for each plot and expressed as Mg C ha^-1^: tree aboveground carbon stock (Tree AGC), estimated tree root carbon stock (Tree RC), shrub aboveground carbon stock (Shrub AGC), and estimated shrub root carbon stock (Shrub RC). This framework resolved woody carbon across two forest strata, including tree and shrub, and two biomass compartments, including aboveground and root. Aboveground and root biomass were estimated for each recorded individual using the allometric equations reported by Wu et al. (2025). Species-specific equations were applied where available; genus- or family-level equations were used otherwise. Individual estimates were summed within plots to derive aboveground and root biomass for each stratum. Biomass was converted to carbon stock using carbon-concentration coefficients of 0.46 for aboveground biomass and 0.44 for root biomass (Schuldt et al., 2023). All estimates were standardized on a per-hectare basis. Because root biomass was derived from allometric equations rather than direct excavation, Tree RC and Shrub RC were treated as allometrically estimated root-carbon stocks. They were retained as corresponding carbon components to examine whether fitted associations were consistent between aboveground and allometrically estimated root allocation within each woody stratum.

### 2.5 Multidimensional plot-level biotic predictors

Plot-level biotic attributes were represented by four complementary dimensions: taxonomic diversity, functional dispersion, dominant trait composition, and structural diversity (Naka and Fatunsin, 2025; Springer et al., 2024). Taxonomic diversity was quantified using the Shannon–Wiener index, calculated from relative species abundances with the vegan package in R. Functional attributes were characterized using six traits associated with resource-use and growth strategies: leaf area, specific leaf area, leaf dry matter content, root volume, specific root length, and root tissue density. Traits were measured following the protocols described by other scholars (Khan et al., 2025; Weigelt et al., 2021; Zhao et al., 2024). Functional dispersion was represented by Rao’s quadratic entropy (Rao’s Q), calculated from the six traits and species-relative abundances. Rao’s Q incorporated both interspecific trait dissimilarity and abundance distributions and was calculated using the FD package in R. Dominant trait composition was summarized using community-weighted mean (CWM) trait values. CWM values were calculated for each plot using relative species abundance as a weighting factor. All CWM variables were log-transformed before principal component analysis (PCA), and the first principal component was retained as an integrated axis of dominant trait composition (Gao et al., 2021; Lin et al., 2016). Structural diversity was characterized using coefficients of variation in DBH (CVd), tree height (CVh), and crown width (CVc). These variables represented complementary aspects of horizontal and vertical stand heterogeneity (Sun et al., 2025; Zhai et al., 2024). The predictors described plot-level community composition and stand structure and were related parallel to each of the four carbon components. They were not interpreted as diversity attributes measured independently for every carbon compartment.

### 2.6 Statistical analyses

All analyses were conducted in R version 4.3.2 (R Core Team, 2023). The analytical workflow comprised three stages: comparison of carbon components and community attributes between fertility contexts; parallel assessment of plot-level predictors; and estimation of context-specific pathway models.

#### 2.6.1 Carbon-component and community comparisons

Tree and shrub aboveground and estimated root carbon stocks were compared between LF and HF plots to determine whether the four components exhibited similar or contrasting distributions across fertility contexts. Species-diversity, functional, trait-compositional and structural variables were compared between the same groups. Student’s t-tests were used when variables satisfied assumptions of normality and homogeneity of variance. Welch’s t-tests were used when normality was supported but variances differed. Wilcoxon rank-sum tests were applied to variables that departed from normality. Transformations were applied where specified in the corresponding analyses and figure captions.

#### 2.6.2 Parallel assessment of plot-level predictors across carbon compartments

Pearson correlations and linear regressions were fitted separately for LF and HF plots and for each woody-carbon component. This parallel compartment-specific framework evaluated whether associations involving species diversity, functional diversity, CWM trait composition, and structural diversity were consistent across Tree AGC, Tree RC, Shrub AGC, and Shrub RC. Relative predictor importance was estimated separately for each fertility context and carbon component using the relaimpo package (Lin et al., 2023; Zheng et al., 2025). Candidate predictors included species diversity, functional diversity, CWM trait composition, structural-diversity metrics, and abiotic variables. These analyses quantified the relative contribution of individual predictors to fitted variation in each carbon component. Contrasts between LF and HF analyses were interpreted descriptively. Separate significance patterns were not treated as formal evidence that coefficients differed between fertility contexts.

#### 2.6.3 Context-specific pathway models

Piecewise structural equation models (SEMs) were fitted separately for LF and HF plots and for each woody-carbon component using the piecewiseSEM package (Lefcheck, 2016). The models evaluated hypothesized direct and indirect associations among abiotic conditions, biodiversity dimensions, structural diversity, and carbon stocks. Abiotic conditions were summarized using the first two principal components of the environmental variables, designated Abiotic PC1 and Abiotic PC2. Abiotic PC1 represented a gradient positively associated with SWC and altitude and was negatively associated with MAT and BD. Abiotic PC2 was positively associated with MAP and MAT and negatively associated with altitude, BD, and pH (**Supplementary Figure S2**). Biotic predictors included functional diversity, species diversity, and structural diversity. Structural diversity was summarized by the first principal component derived from CVd, CVh, and CVc (**Supplementary Figure S3**). The initial SEM structure was specified from *a priori* ecological hypotheses (**Supplementary Figure S4**), and competing models were compared using Akaike’s information criterion corrected for small sample size (AICc). Model adequacy was evaluated using d-separation tests based on Fisher’s C statistic. Values of P > 0.05 indicated that the implied conditional-independence claims were not rejected. Among models meeting this criterion, the model with the lowest AICc was retained as the best-supported model. Differences among separately fitted SEMs were interpreted descriptively as contrasts in pathway direction, coefficient magnitude, predictor configuration, and explained variance across carbon components and fertility contexts. They were not treated as formal tests of between-group or between-compartment coefficient differences.

## 3 Results

### 3.1 Four woody-carbon components showed contrasting patterns across fertility contexts

The four woody-carbon components differed between LF (n = 33) and HF (n = 21) contexts, but the direction of the difference varied by forest stratum. Tree aboveground carbon was higher in LF than in HF plots (*P* = 0.043), as was estimated tree root carbon (*P* = 0.033; **Figures 2a, b**). By contrast, shrub aboveground carbon (*P* = 0.027) and estimated shrub root carbon (*P* = 0.004) were higher in HF plots (**Figures 2c, d**). Thus, carbon stocks varied concordantly within strata but in opposite directions between tree and shrub strata. Aboveground and estimated root carbon were strongly positively correlated within each stratum (**Supplementary Figure S5**). In LF plots, the correlations were r = 0.96 for tree carbon and r = 0.90 for shrub carbon; in HF plots, the corresponding values were r = 0.95 and r = 0.69 (all *P* < 0.001). By contrast, the tree and shrub carbon components were negatively correlated. Cross-stratum correlations ranged from r = −0.81 to −0.90 in LF plots and from r = −0.62 to −0.84 in HF plots (all *P* < 0.01; **Supplementary Figure S5**). The two fertility groups had similar mean altitude and stand age. The mean altitude was 730.33 ± 212.58 m in LF plots and 718.67 ± 124.93 m in HF plots, whereas the mean stand age was 32.39 ± 4.99 and 31.38 ± 5.42 years, respectively. Stand density was descriptively higher in HF plots (1374 ± 233 trees ha^-1^) than in LF plots (1168 ± 208 trees ha^-1^). Mean DBH and tree height were descriptively higher in LF plots (17.50 ± 6.85 cm and 11.53 ± 2.98 m) than in HF plots (15.53 ± 6.69 cm and 10.02 ± 2.90 m; **Supplementary Table S1**). Plot-level biotic attributes provided additional context for these carbon-stock differences.

**Figure 2:**
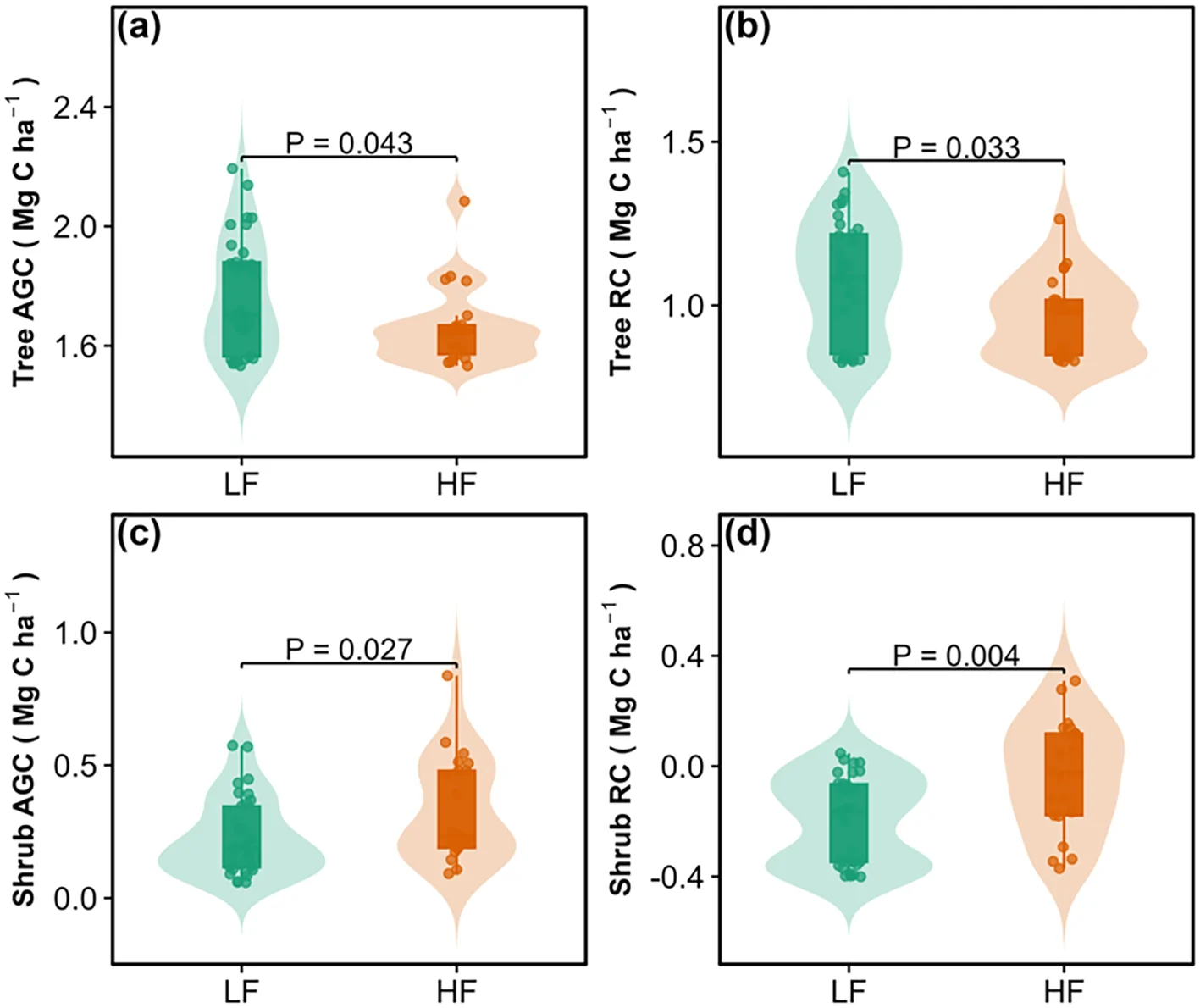
Carbon stocks differed between fertility contexts in opposite directions across forest strata. Differences in **(a)** tree aboveground carbon stock (Tree AGC), **(b)** estimated tree root carbon stock (Tree RC), **(c)** shrub aboveground carbon stock (Shrub AGC) and **(d)** estimated shrub root carbon stock (Shrub RC) between low-fertility (LF) and high-fertility (HF) plots. Variables were log-transformed before analysis.

Shannon diversity did not differ between LF and HF plots (*P* = 0.556; **Figure 3a**), whereas community-weighted mean (CWM) trait scores (*P* = 0.010) and functional diversity (FD; *P* = 0.029) were higher in HF plots (**Figures 3b, c**). Structural-diversity metrics did not differ between contexts: CVd, *P* = 0.523; CVh, *P* = 0.380; and CVc, *P* = 0.632 (**Figures 3d–f**). CWM trait scores and FD were strongly correlated in both LF (r = 0.82, *P* < 0.001) and HF plots (r = 0.76, *P* < 0.001). CVd and CVh were also positively correlated in LF (r = 0.65, *P* < 0.001) and HF plots (r = 0.84, *P* < 0.001; **Supplementary Figure S6**).

**Figure 3:**
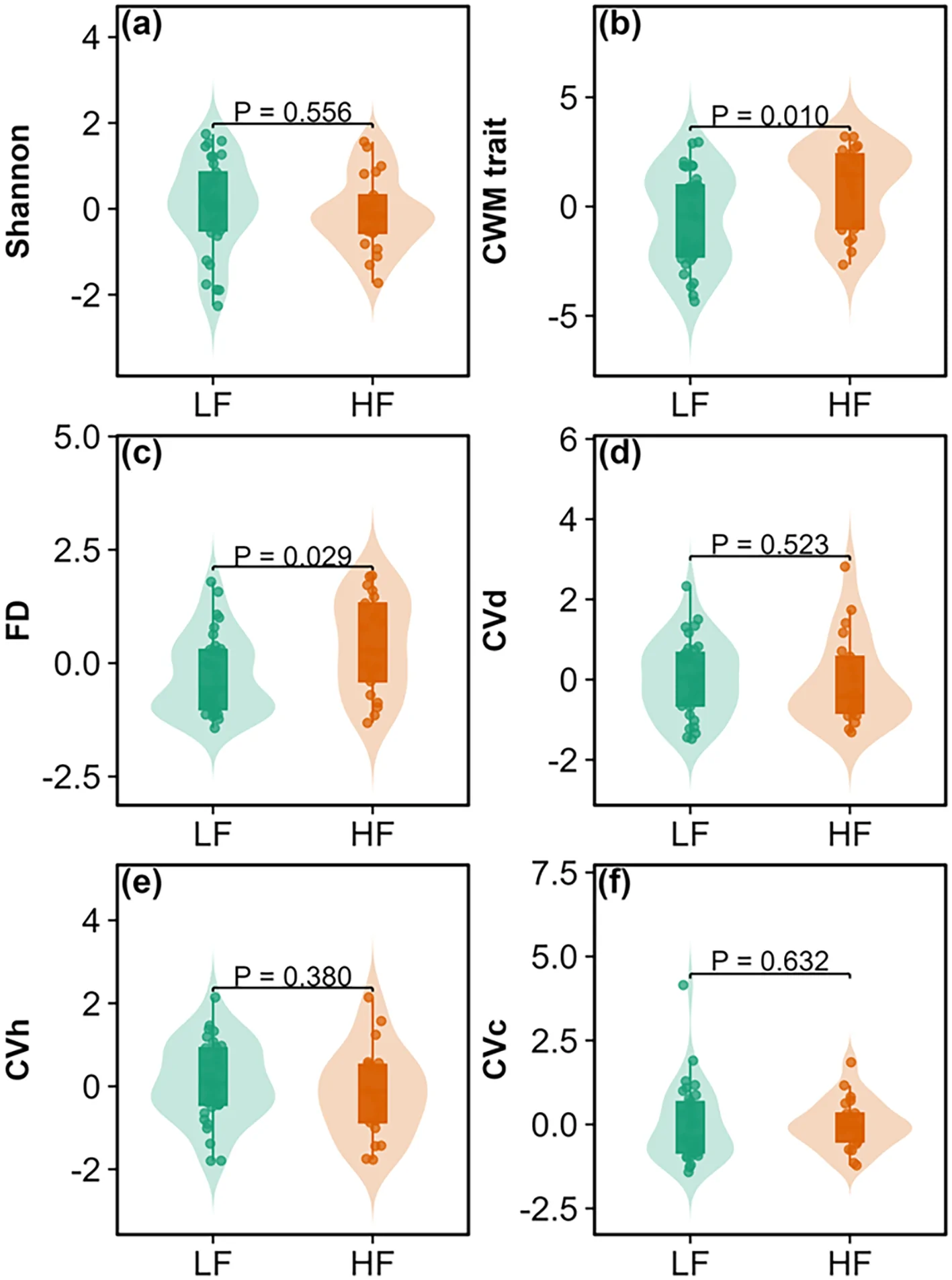
Plot-level diversity and structural attributes across fertility contexts. Differences in **(a)** Shannon diversity, **(b)** community-weighted mean trait score (CWM trait), **(c)** functional diversity (FD), **(d)** coefficient of variation in diameter at breast height (CVd), **(e)** coefficient of variation in tree height (CVh) and **(f)** coefficient of variation in crown width (CVc) between low-fertility (LF) and high-fertility (HF) plots. Variables were standardized using z-scores before analysis.

### 3.2 Multidimensional predictor associations revealed cross-stratum contrasts

Bivariate associations between the same plot-level biotic attributes and carbon stocks varied across carbon components and fertility contexts (**Figure 4**; **Supplementary Figure S7**). The strongest cross-stratum contrast occurred in HF plots. Shannon diversity was positively related to tree aboveground carbon (R² = 0.21, *P* = 0.037; **Figure 4i**) and estimated tree root carbon (R² = 0.25, *P* = 0.021; **Figure 4l**), but negatively related to shrub aboveground carbon (R² = 0.19, *P* = 0.049; **Figure 4d**) and estimated shrub root carbon (R² = 0.32, *P* = 0.008; **Figure 4h**). Structural attributes showed a comparable cross-stratum pattern. In HF plots, the estimated tree root carbon was positively related to CVd (R² = 0.21, *P* = 0.038) and CVh (R² = 0.27, *P* = 0.016; **Figures 4j, k**). Shrub aboveground carbon was negatively related to both CVd (R² = 0.31, *P* = 0.009) and CVh (R² = 0.38, *P* = 0.003; **Figures 4a, b**), and the estimated shrub root carbon was negatively related to CVh (R² = 0.21, *P* = 0.039; **Figure 4e**). FD was negatively related to shrub aboveground carbon in HF plots (R² = 0.21, *P* = 0.036; **Figure 4c**) but was unrelated to estimated shrub root carbon (R² = 0.01, *P* = 0.682; **Figure 4g**). CWM trait composition was also unrelated to estimated shrub root carbon under HF conditions (R² = 0.03, *P* = 0.491; **Figure 4f**). Fewer significant bivariate relationships occurred in LF plots. Estimated shrub root carbon was positively related to CWM trait composition (R² = 0.19, *P* = 0.011; **Figure 4f**) and FD (R² = 0.14, *P* = 0.034; **Figure 4g**).

**Figure 4:**
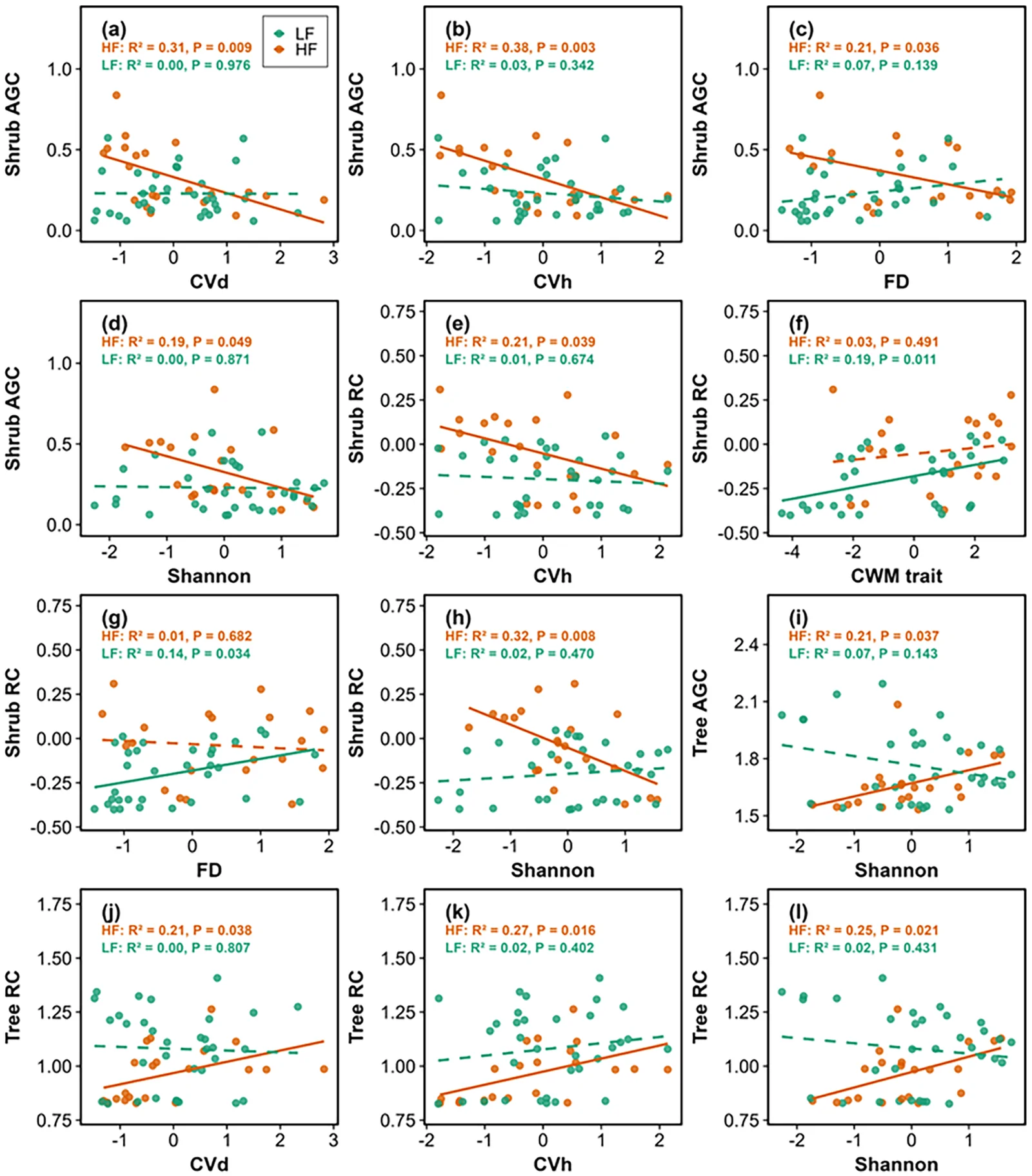
Diversity–carbon associations differed across woody compartments and fertility contexts. Relationships of **(a–d)** shrub aboveground carbon with CVd, CVh, FD and Shannon diversity; **(e–h)** estimated shrub root carbon with CVh, CWM trait composition, FD and Shannon diversity; **(i)** tree aboveground carbon with Shannon diversity; and **(j–l)** estimated tree root carbon with CVd, CVh and Shannon diversity. Models were fitted separately for low-fertility (LF) and high-fertility (HF) plots. Solid lines denote significant regressions and dashed lines denote non-significant regressions. Panel annotations report R² and *P* values.

The remaining relationships shown in **Figure 4** were not significant under LF conditions. These included Shannon diversity with tree aboveground carbon (R² = 0.07, *P* = 0.143) and estimated tree root carbon (R² = 0.02, *P* = 0.431) and CVd and CVh with estimated tree root carbon (R² ≤ 0.02, *P* ≥ 0.402). Across the separately fitted regressions, the same plot-level diversity or structural attribute was therefore not related consistently to carbon across forest strata. In HF plots, Shannon diversity and tree-size heterogeneity were positively related to tree carbon components but negatively related to shrub carbon components.

### 3.3 Predictor importance and fitted pathway configurations varied among carbon compartments

Relative-importance analyses yielded distinct predictor rankings across carbon components and fertility contexts (**Figure 5**). The fitted models accounted for more variation in HF than in LF plots: R² = 0.82 versus 0.32 for Tree AGC, 0.84 versus 0.33 for Tree RC, 0.80 versus 0.29 for Shrub AGC and 0.84 versus 0.33 for Shrub RC (**Figures 5a–d**). For Tree AGC, FD and CVc had the largest relative contributions in LF plots, whereas Shannon diversity, slope, and MAT ranked highest in HF plots (**Figure 5a**). For Tree RC, CVc, MAP, and FD contributed most in LF plots, whereas Shannon diversity, MAT, slope, CVh and altitude ranked highest in HF plots (**Figure 5b**). MAP accounted for the largest share of explained variation in Shrub AGC under LF conditions, followed by CWM trait composition and FD; under HF conditions, CVh, Shannon diversity, slope, and MAT ranked highest (**Figure 5c**). For Shrub RC, CWM trait composition, MAP, and FD were the principal LF predictors, whereas Shannon diversity ranked highest under HF conditions, followed by slope, CVh, and CWM trait composition (**Figure 5d**).

**Figure 5:**
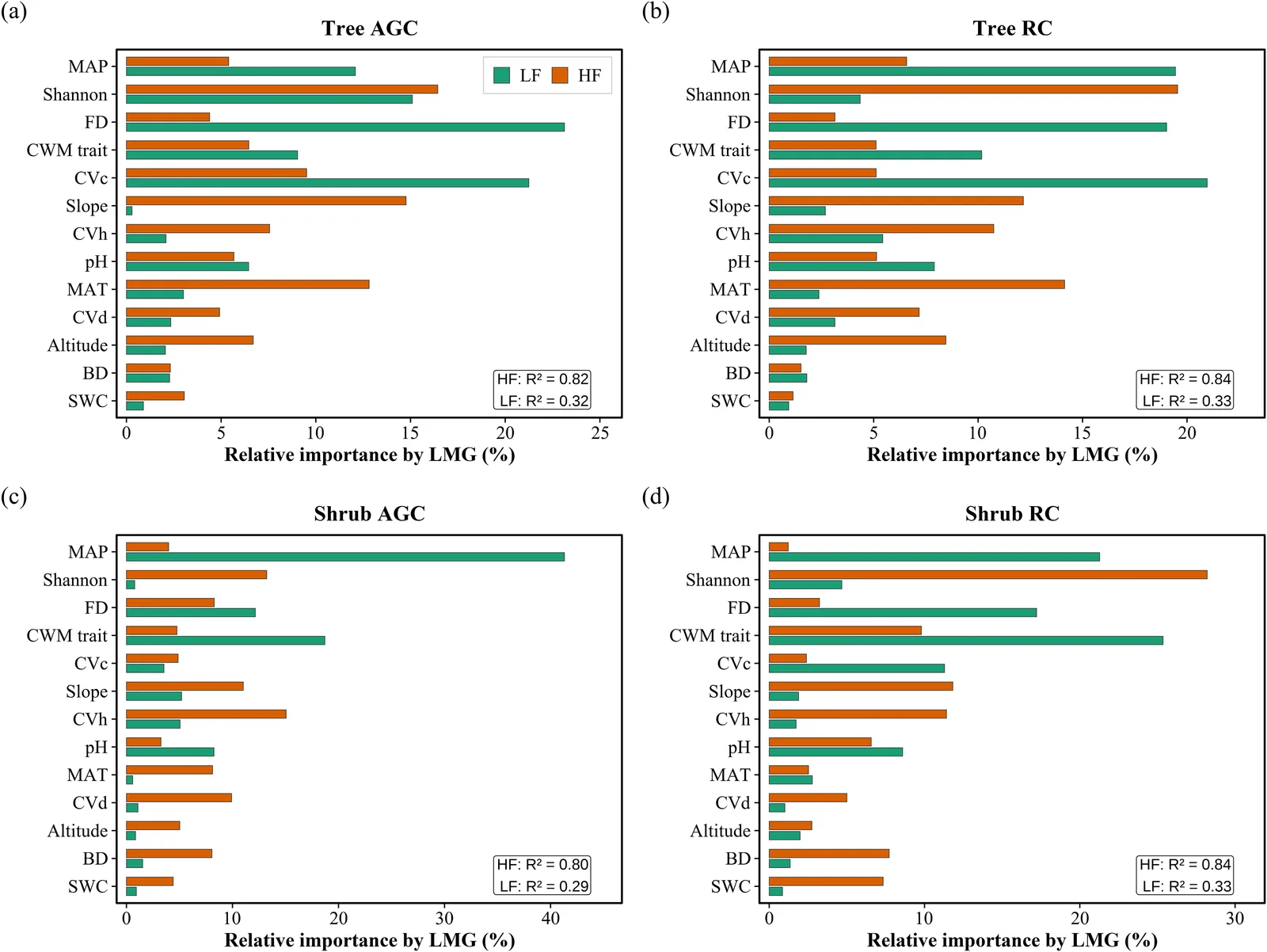
Predictor importance differed among carbon components and fertility contexts. Relative importance of plot-level diversity, structural and environmental predictors for **(a)** tree aboveground carbon, **(b)** estimated tree root carbon, **(c)** shrub aboveground carbon and **(d)** estimated shrub root carbon under low-fertility (LF) and high-fertility (HF) conditions. Relative importance was quantified using the LMG metric. Green and orange bars denote LF and HF plots, respectively.

All eight piecewise structural equation models satisfied the d-separation criterion (Fisher’s C = 1.62–3.75, *P* = 0.88–0.95; **Figure 6**). The HF models accounted for 62% of the variation in Tree AGC, 73% in Tree RC, 67% in Shrub AGC, and 58% in Shrub RC (**Figures 6a, c, e, g**). The corresponding LF models accounted for 19%, 17%, 16%, and 18%, respectively (**Figures 6b, d, f, h**). In the HF tree models, Abiotic PC1 was negatively associated with Tree AGC (β = −0.47, *P* < 0.05) and Tree RC (β = −0.59, *P* < 0.01), and Abiotic PC2 was negatively associated with the same components (β = −0.37 and −0.36, respectively; both *P* < 0.05) (**Figures 6a, c**). FD was negatively associated with Tree AGC (β = −0.58, *P* < 0.05). By contrast, species diversity was positively associated with Tree AGC (β = 0.47, *P* < 0.05) and Tree RC (β = 0.55, *P* < 0.01). The HF shrub models showed the opposite species-diversity pattern. Species diversity was negatively associated with Shrub AGC (β = −0.51, *P* < 0.05) and Shrub RC (β = −0.70, *P* < 0.01; **Figures 6e, g**). Abiotic PC1 was positively associated with Shrub AGC (β = 0.60, *P* < 0.01) and Shrub RC (β = 0.55, *P* < 0.05). Direct pathways from FD and structural diversity to shrub carbon were not significant. Under LF conditions, FD was the only biotic predictor with significant direct paths to all four carbon components, but the coefficient direction differed between strata. FD was negatively associated with Tree AGC (β = −0.38, *P* < 0.05) and Tree RC (β = −0.39, *P* < 0.05), but positively associated with Shrub AGC (β = 0.38, *P* < 0.05) and Shrub RC (β = 0.43, *P* < 0.05; **Figures 6b, d, f, h**). Species-diversity and structural-diversity paths to the LF carbon components were not significant. The fitted models therefore converged on two directional cross-stratum contrasts: species-diversity coefficients were positive for tree carbon and negative for shrub carbon in HF plots, whereas FD coefficients were negative for tree carbon and positive for shrub carbon in LF plots.

**Figure 6:**
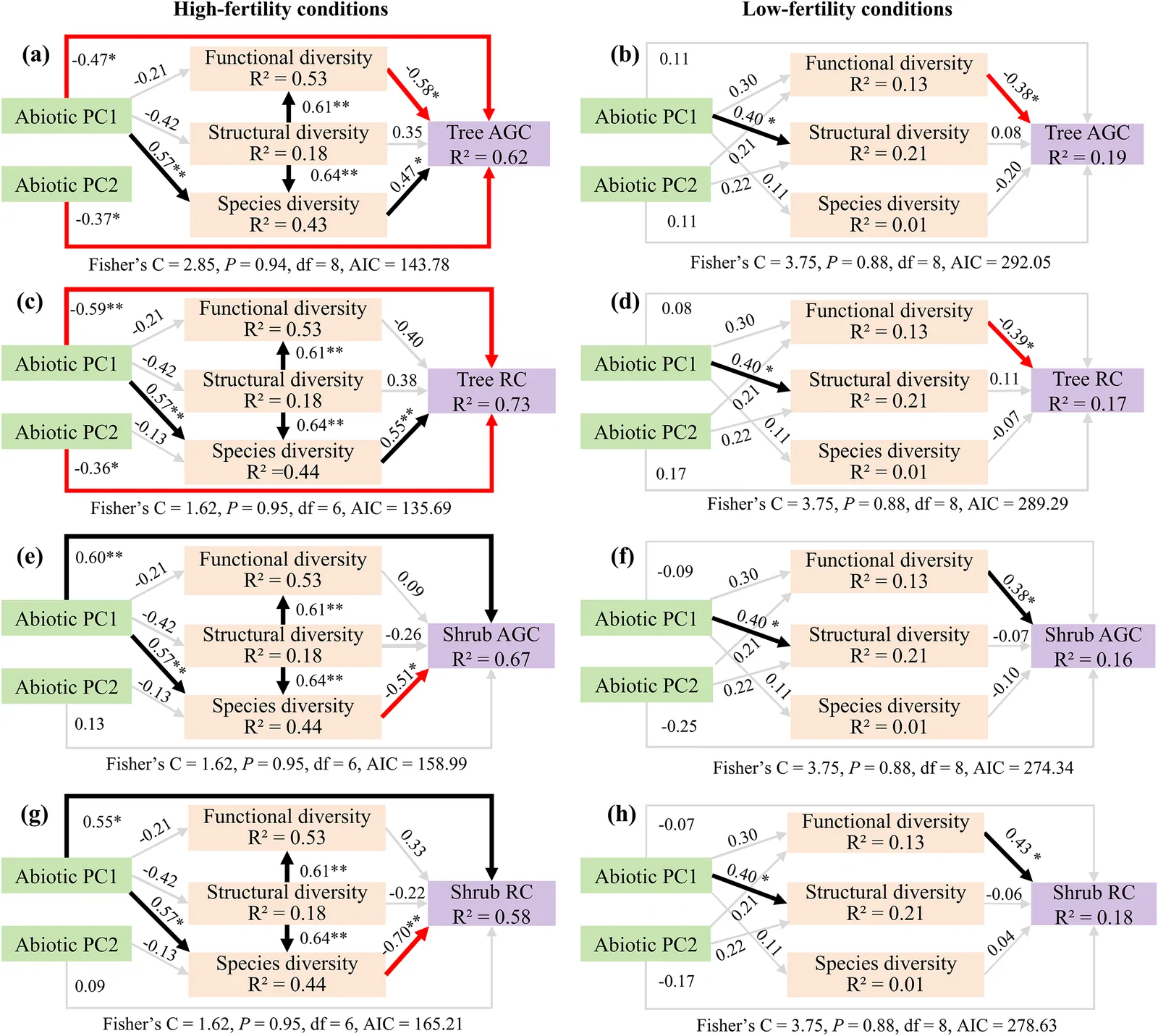
Fitted pathway configurations differed across woody-carbon components and fertility contexts. Piecewise structural equation models of hypothesized direct and indirect associations among abiotic conditions, biodiversity dimensions, structural diversity and carbon stocks. Panels correspond to **(a, b)** Tree AGC, **(c, d)** Tree RC, **(e, f)** Shrub AGC and **(g, h)** estimated Shrub RC under high-fertility (HF) and low-fertility (LF) conditions. Arrow width is proportional to the absolute standardized path coefficient. Black and red arrows denote significant positive and negative associations, respectively; grey arrows denote non-significant paths. R² denotes variance accounted for in endogenous variables. Significance levels are *P* < 0.05 (*) and *P* < 0.01 (**). Fisher’s *C*, *P*, degrees of freedom and information-criterion values are reported beneath each model.

## 4 Discussion

The four woody carbon components did not vary uniformly across fertility contexts. Tree aboveground and estimated root carbon were higher in LF plots, whereas shrub aboveground and estimated root carbon were higher in HF plots. Predictor directions also differed across strata: species diversity was positively associated with tree carbon but negatively associated with shrub carbon under HF conditions, whereas functional diversity showed negative paths to tree carbon and positive paths to shrub carbon under LF conditions. Relative predictor importance and fitted SEM configurations likewise differed among carbon components and fertility contexts. Together, these results indicated that aggregated carbon metrics can conceal ecologically meaningful cross-stratum and cross-pool heterogeneity (Lin et al., 2026; Lunow et al., 2026).

### 4.1 Compartment resolution reveals contrasting tree–shrub carbon patterns

The opposing tree–shrub patterns were consistent with vertical differentiation in woody carbon organization rather than a uniform association with soil fertility. The higher tree carbon observed in LF plots may reflect larger individuals, stronger overstory dominance, stand history, or dominant species composition; mean DBH and tree height were also somewhat greater in LF plots (**Supplementary Table S1**). Large trees commonly account for a disproportionate fraction of woody biomass because of their size, canopy residence time, and access to deeper soil resources (Soong et al., 2020). The observed pattern, therefore, did not indicate that low fertility favored tree growth. Instead, it was compatible with structural and compositional differences between plots and with previous evidence that overstory and understory biomass can show distinct environmental associations (Zhang et al., 2023b). The higher shrub carbon in HF plots may have corresponded with the greater availability of near-surface nutrients, although understory biomass is also closely related to canopy light, shallow-root competition, and local stand structure (Ali et al., 2019; Zhang et al., 2017). HF plots may have also contained community configurations with fewer large trees, greater shrub abundance, or different dominant taxa (Bolstad et al., 2018; Li et al., 2026b). Positive above-ground-root correlations within strata and negative tree–shrub correlations (**Supplementary Figure S5**) documented coordinated within-stratum variation and cross-sectional opposition; they did not indicate carbon transfer between layers. Together, these results reinforce the need to distinguish carbon stored in different woody strata when evaluating forest carbon responses to environmental variation (Wu et al., 2025; Chen et al., 2025; Zhou et al., 2026).

### 4.2 Biodiversity–carbon associations are dimension- and compartment-specific

Plot-level species diversity showed the clearest directional contrast under HF conditions: it was positively associated with tree above-ground and estimated root carbon but negatively associated with both shrub components. The positive tree associations were consistent with stand-level patterns potentially arising from selection and complementarity interpretations, whereby species-rich stands may contain high-biomass taxa or exhibit differentiated canopy positions, rooting profiles, and resource-use strategies (Chen et al., 2024). Comparable relationships have been reported where community composition and niche differentiation corresponded to greater trees biomass (Jing et al., 2021; Li et al., 2019; Zheng et al., 2025). The negative shrub associations may instead reflect recruitment of numerous small individuals, compositional turnover, or dilution of biomass concentrated in dominant shrubs. In dense understories, greater richness can coexist with lower mean individual biomass, while size inequality may correspond to asymmetric access to light and space (Bolstad et al., 2018; Bourdier et al., 2016). These explanations remain plausible interpretations rather than processes isolated by the present design.

Plot-level functional diversity also showed contrasting associations with carbon stored in different strata. Under LF conditions, the SEM coefficients were negative for tree carbon but positive for shrub carbon, while CWM trait composition and functional diversity were positively related to estimates of shrub root carbon in the bivariate analyses. In resource-limited settings, functional differentiation among shrubs may correspond to variation in root morphology, nutrient acquisition, and conservative resource-use strategies (Augusto and Boča, 2022; Deng et al., 2023; Zhao et al., 2024). This pattern was consistent with hypotheses involving variation in belowground resource-use strategies and dominant trait composition (Gao et al., 2021; Lin et al., 2016; Weigelt et al., 2021), although neither interpretation was directly tested and root carbon was estimated allometrically rather than measured directly. Similar associations between trait composition, functional diversity, and belowground carbon have been reported elsewhere (Guo et al., 2025). Structural diversity showed analogous compartment dependence. Positive relationships of diameter and height variation with estimated tree root carbon were compatible with differentiated space occupation, the presence of large individuals, or variation in rooting profiles (Fatunsin, 2025; Noulèkoun et al., 2024). By contrast, negative relationships with shrub carbon may have corresponded with canopy restriction, size inequality, or crowding beneath structurally heterogeneous overstory stands (Guo et al., 2025; Zhai et al., 2024). Thus, structural variation that coincided with greater tree carbon was not necessarily associated with greater understory carbon.

### 4.3 Fertility context differentiates biotic and abiotic predictor configurations

The measured predictor set accounted for substantially more observed variance under HF conditions. SEM (R²) values ranged from 0.58 to 0.73 in HF plots but from only 0.16 to 0.19 in LF plots (**Figure 6**). Abiotic PCs were associated negatively with tree carbon and positively with shrub carbon in the HF models, while the dominant predictors identified by relative-importance analysis differed across all four components (**Figure 5**). The higher explanatory power under HF conditions may have reflected more coherent resource gradients, less unmeasured heterogeneity, or subgroup-specific covariance structures. Conversely, carbon variation in the LF plot may have corresponded more strongly to factors not represented in the fitted models, including stand history, species identity, disturbance, water availability, or deeper soil resources (Hwang and Zhai, 2026; Lanuza et al., 2026; Lin et al., 2023). The biotic pathways reinforce this contextual heterogeneity. Under HF conditions, positive species-diversity paths to tree carbon contrasted with negative paths to shrub carbon. Under LF conditions, functional diversity paths were negative for tree carbon but positive for shrub carbon. Related overstory–understory contrasts have been reported where vegetation strata differ in resource access and biomass organization (Chen et al., 2025; Ma et al., 2023). In tree communities, greater species diversity may coincide with the inclusion of high-biomass taxa or differentiated resource-use strategies (Gao et al., 2021; Jing et al., 2021). In shrub communities, greater diversity may instead coincide with small-individual recruitment or reduced dominance by high-biomass taxa (Sonkoly et al., 2019). Likewise, functional diversity under nutrient limitations need not carry a common association across strata (Luo et al., 2019b).

### 4.4 Implications for carbon assessment and limitations

Tree aboveground carbon alone did not represent the full woody-carbon pattern. Shrub and estimated root components showed different, and sometimes opposing, associations with biodiversity, stand structure, and abiotic conditions. Similar cross-stratum contrasts have also been reported in non-karst forests (Ali et al., 2019; Zhang et al., 2023b). For example, Chen et al. (2025) found in a subtropical forest biodiversity experiment that increasing tree species richness promoted tree biomass but reduced shrub biomass, highlighting contrasting biodiversity–biomass responses between overstory and understory strata. Carbon assessments would therefore benefit from identifying the target stratum and pool explicitly, because whole-community aggregation can conceal sign reversals and shifts in predictor importance (Haq et al., 2024). Management objectives could likewise specify the carbon component of interest rather than assume that biodiversity interventions correspond similarly to every forest layer. Fertility contexts may inform expectations, but the presented evidence did not prescribe nutrient manipulation or stratum-specific treatment (Guo et al., 2025; Zhai et al., 2024). Several limitations constrained inference. First, the observational design identified associations rather than causal relationships. Second, the separately fitted LF and HF models did not constitute formal interaction tests; differences in statistical significance therefore did not establish differences between coefficients. Third, subgroup sizes were small and unequal, leaving the SEM estimates potentially sensitive to influential observations. Fourth, most predictors, including biodiversity and trait-composition metrics, were quantified at the plot level rather than separately for tree and shrub strata. Thus, cross-stratum contrasts reflect associations of the same plot-level biotic gradients with stratum-specific carbon stocks, while future stratum-specific diversity assessments are needed to test whether comparable directional contrasts occur within each woody layer. Fifth, LF plots contained somewhat larger and taller trees, so stand structure, history, and species composition may have covaried with fertility classification. Sixth, fertility was derived from a continuous multivariate gradient and may have covaried with water availability, topography, and light (Zhang et al., 2023a). Finally, shrub biomass and all root-carbon components were estimated using allometric equations and fixed carbon-conversion coefficients. Because aboveground and estimated root carbon were derived from the same recorded individuals, these components were inherently correlated and should not be interpreted as statistically independent evidence of aboveground versus belowground ecological processes. Their strong correspondence, particularly in the tree stratum, may partly reflect shared plant-size information and the structure of the allometric equations. Direct root measurements would be required to evaluate belowground biodiversity–carbon relationships independently.

## 5 Conclusion

We resolved woody carbon into four corresponding components: tree aboveground carbon, allometrically estimated tree root carbon, shrub aboveground carbon, and allometrically estimated shrub root carbon. We evaluated each component using a common multidimensional predictor framework across low- and high-fertility plots. Carbon stocks did not vary uniformly between fertility contexts: tree carbon was higher in low-fertility plots, whereas shrub carbon was higher in high-fertility plots. Predictor identities and directions likewise differed among compartments. Under high-fertility conditions, plot-level species diversity was positively associated with tree carbon but negatively associated with shrub carbon; under low-fertility conditions, plot-level functional diversity showed negative associations with tree carbon and positive associations with shrub carbon. Structural and abiotic predictors also differed in relative importance across strata, pools, and fertility contexts. These contrasts show that biodiversity-carbon relationships inferred from tree aboveground biomass or an aggregated forest-carbon metric may not represent the full organization of woody carbon in vertically stratified forests. Explicit resolution of forest stratum, carbon pool, and environmental context, therefore, provides a more precise basis for interpreting biodiversity–carbon associations. Incorporating understory woody vegetation and estimated root components into forest-carbon assessments may reveal ecological patterns that remain concealed when carbon stocks are treated as a single community-level response. For karst forests, restoration and carbon-management strategies should explicitly account for their strong soil-resource heterogeneity by considering both overstory trees and understory shrubs and maintaining vertical structural complexity.

## Data Availability

Publicly available datasets were analyzed in this study. This data can be found here: The data supporting this study are available in the Figshare repository at https://doi.org/10.6084/m9.figshare.27979243.v1 (Gao et al., 2025) and https://doi.org/10.6084/m9.figshare.27308826 (Wu et al., 2025).
