# Supplementary material for: Soil fertility context shapes biodiversity–carbon associations across forest strata in a subtropical karst landscape
Source: Front Plant Sci. 2026 Sep 23;17:1942459. doi: 10.3389/fpls.2026.1942459 (PMC13642828; doi:10.3389/fpls.2026.1942459)
Supplement: Supplementary file 1 [file DataSheet1.docx]

**Supplementary Material**

**Table S1**. Summary of plot-level stand characteristics under high- and low-fertility conditions, including sample size, altitude, forest age, diameter at breast height, tree height, and stand density.

| Fertility | Sample size | Altitude | Forest age (years) | Diameter at breast height (DBH, cm) | Tree height (m) | Stand density (trees/ha) |
| --- | --- | --- | --- | --- | --- | --- |
| High-fertility | 21 | 718.67±124.93 | 31.38±5.42 | 15.53±6.69 | 10.02±2.9 | 1374±233 |
| Low-fertility | 33 | 730.33±212.58 | 32.39±4.99 | 17.5±6.85 | 11.53±2.98 | 1168±208 |

**Fig. S1**. Hierarchical clustering dendrogram used to classify forest plots into low- and high-fertility groups. Each terminal label represents an individual forest plot. Branch colors indicate the two fertility groups identified by the clustering analysis: low fertility in green and high fertility in orange.

**Fig. S2. Principal component analysis (PCA) of environmental variables under low- and high-fertility conditions.** (a) PCA ordination of plot-level environmental conditions, with points representing individual plots and ellipses showing the distribution of low-fertility (LF) and high-fertility (HF) plots. PC1 and PC2 explained 43.7% and 18.6% of the total variation, respectively. (b) Correlation circle showing the direction and strength of environmental variable loadings on PC1 and PC2. (c) Relative contributions of each environmental variable to PC1 and PC2. (d) Differences in PCA scores between low- and high-fertility plots, assessed for PC1 and PC2. MAT, mean annual temperature; MAP, mean annual precipitation; SWC, soil water content; BD, soil bulk density.

**Fig. S3. Principal component analysis (PCA) of stand structural diversity under low- and high-fertility conditions.** (a) PCA ordination based on three structural diversity metrics, including CVd, CVh and CVc. Points represent individual forest plots, and ellipses indicate the distribution of low-fertility (LF) and high-fertility (HF) plots. PC1 and PC2 explained 59.3% and 32.0% of the total variation, respectively. (b) Correlation circle showing the direction and strength of variable loadings on the first two PCA axes. (c) Relative contribution of each structural diversity variable to PC1 and PC2. (d) Comparison of PCA scores between LF and HF plots. Numbers above brackets indicate P values. CVd, CVh and CVc represent structural variation in diameter, height and crown-related attributes, respectively.

**Fig. S4. Hypothesized piecewise structural equation model illustrating how abiotic variables, represented by the first two principal components (abiotic PCA1 and PCA2), and multidimensional biodiversity, including species diversity, functional diversity, and structural diversity, directly or indirectly influence carbon stock.**

**Fig. S5. Pearson correlations among tree and shrub aboveground and root carbon stocks.** Panels represent correlations for (a) all plots, (b) low-fertility plots and (c) high-fertility plots. Red and blue colors indicate positive and negative correlations, respectively, with color intensity proportional to the Pearson correlation coefficient. Numbers in cells indicate Pearson’s r. Tree AGC, tree aboveground carbon stock; Tree RC, tree root carbon stock; Shrub AGC, shrub aboveground carbon stock; Shrub RC, shrub root carbon stock. Significance levels are indicated as **P < 0.01 and ***P < 0.001.

**Fig. S6. Pearson correlations among taxonomic, functional and structural diversity variables.** Panels represent correlation matrices for (a) all plots, (b) low-fertility plots and (c) high-fertility plots. Red and blue colors indicate positive and negative correlations, respectively, with color intensity proportional to the Pearson correlation coefficient. Numbers in cells indicate Pearson’s r. Shannon represents species diversity; CWM trait represents community-weighted mean trait composition; FD represents functional diversity; CVd, CVh and CVc represent stand structural diversity in diameter, height and crown-related attributes, respectively. Significance levels are indicated as *P < 0.05, **P < 0.01 and ***P < 0.001.

**Fig. S7. Relationships between tree and shrub carbon stocks and selected functional and structural diversity variables under low- and high-fertility conditions.** Linear regressions were fitted separately for low-fertility (LF, green) and high-fertility (HF, orange) plots. Tree AGC, tree aboveground carbon stock; Tree RC, tree root carbon stock; Shrub AGC, shrub aboveground carbon stock; Shrub RC, shrub root carbon stock; CWM trait, community-weighted mean trait composition; FD, functional diversity; CVd, diameter structural diversity; CVh, height structural diversity; CVc, crown-related structural diversity.
